# Supplementary figures and images for: Evaluating tubulointerstitial compartments in renal biopsy specimens using a deep learning-based approach for classifying normal and abnormal tubules
Source: PLoS One. 2022 Jul 11;17(7):e0271161. doi: 10.1371/journal.pone.0271161 (PMC9273082; doi:10.1371/journal.pone.0271161)

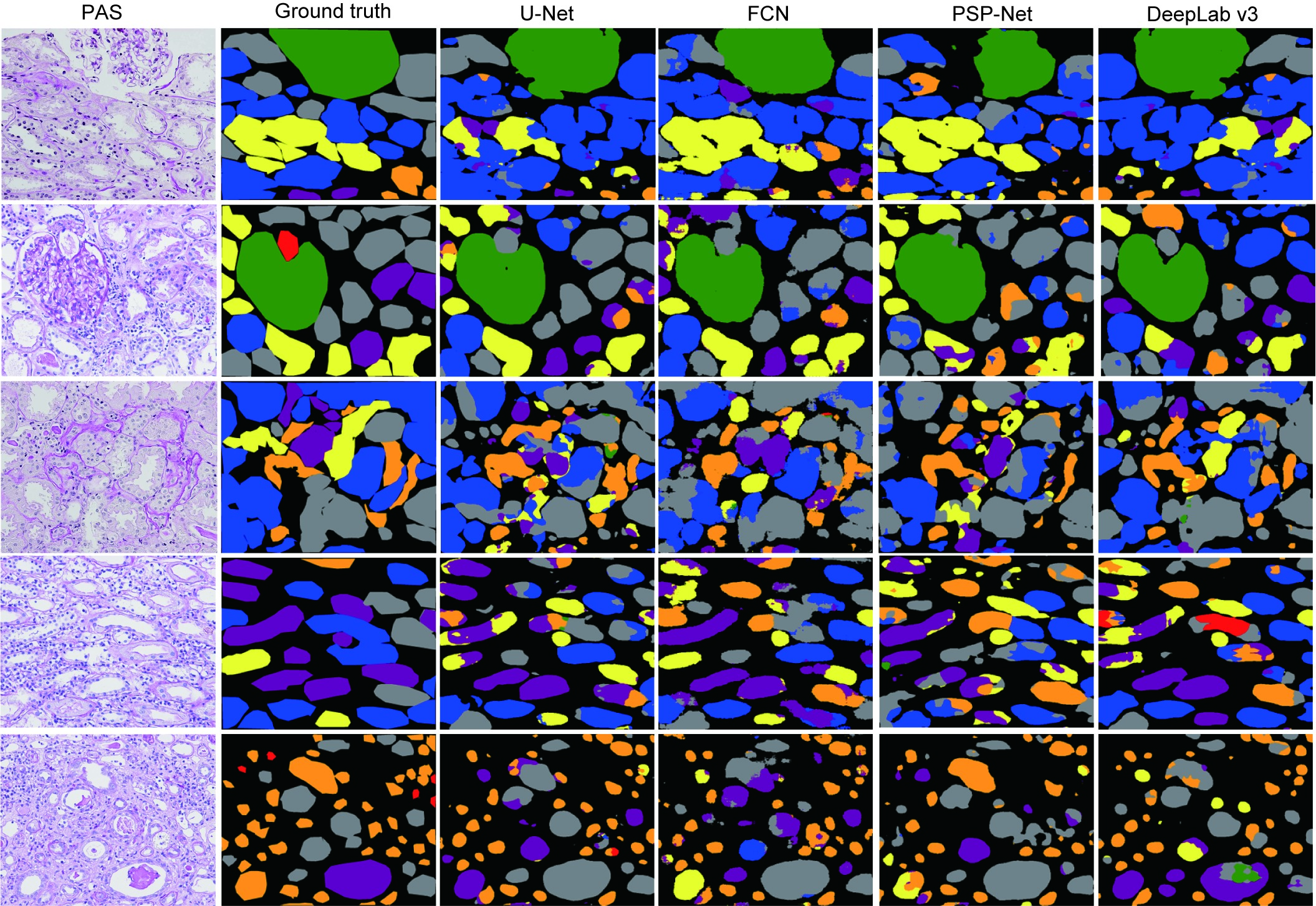

Supplement: S1 Fig — PAS-stained slide, ground truth, and segmentation using U-Net. The top row represents a normal specimen, and the second through fourth rows represent specimens with tubulointerstitial nephritis. (TIF) [file pone.0271161.s001.tif]
