# Supplementary material for: Evaluating tubulointerstitial compartments in renal biopsy specimens using a deep learning-based approach for classifying normal and abnormal tubules
Source: PLoS One. 2022 Jul 11;17(7):e0271161. doi: 10.1371/journal.pone.0271161 (PMC9273082; doi:10.1371/journal.pone.0271161)
Supplement: S1 Table — (DOCX) [file pone.0271161.s002.docx]

**S1 Table. Dice coefficients of various deep learning methods.**

|  | U-Net | FCN | PSP-Net | DeepLab v3 |
| --- | --- | --- | --- | --- |
| Interstitium | 0.85 | 0.85 | 0.83 | 0.84 |
| Glomeruli | 0.90 | 0.91 | 0.93 | 0.94 |
| Proximal tubules | 0.63 | 0.65 | 0.60 | 0.65 |
| Distal tubules | 0.62 | 0.64 | 0.58 | 0.52 |
| Arteries | 0.59 | 0.47 | 0.34 | 0.41 |
| Tubulitis | 0.36 | 0.35 | 0.23 | 0.33 |
| Degenerated tubules | 0.51 | 0.53 | 0.50 | 0.48 |
| Atrophied tubules | 0.46 | 0.46 | 0.44 | 0.43 |
| Average | 0.62 | 0.61 | 0.55 | 0.58 |
